# Amyloid Fibrils of *Pisum sativum* L. Vicilin Inhibit Pathological Aggregation of Mammalian Proteins

**DOI:** 10.3390/ijms241612932

**Published:** 2023-08-18

**Authors:** Maksim I. Sulatsky, Mikhail V. Belousov, Anastasiia O. Kosolapova, Ekaterina V. Mikhailova, Maria N. Romanenko, Kirill S. Antonets, Irina M. Kuznetsova, Konstantin K. Turoverov, Anton A. Nizhnikov, Anna I. Sulatskaya

**Affiliations:** 1 Institute of Cytology, Russian Academy of Sciences, 194064 St. Petersburg, Russia; m_sulatsky@mail.ru (M.I.S.); 4evamkh@gmail.com (E.V.M.); imk@incras.ru (I.M.K.); kkt@incras.ru (K.K.T.); 2 All-Russia Research Institute for Agricultural Microbiology, 196608 St. Petersburg, Russia; belousovmix@gmail.com (M.V.B.); kosolapova97@mail.ru (A.O.K.); romanenkomariabio@gmail.com (M.N.R.); k.antonets@arriam.ru (K.S.A.); 3 Faculty of Biology, St. Petersburg State University, 199034 St. Petersburg, Russia

**Keywords:** vicilin, amyloid, protein fibril, lysozyme, insulin, beta-2-microglobulin, β-amyloid peptide, *Pisum sativum* L.

## Abstract

Although incurable pathologies associated with the formation of highly ordered fibrillar protein aggregates called amyloids have been known for about two centuries, functional roles of amyloids have been studied for only two decades. Recently, we identified functional amyloids in plants. These amyloids formed using garden pea *Pisum sativum* L. storage globulin and vicilin, accumulated during the seed maturation and resisted treatment with gastric enzymes and canning. Thus, vicilin amyloids ingested with food could interact with mammalian proteins. In this work, we analyzed the effects of vicilin amyloids on the fibril formation of proteins that form pathological amyloids. We found that vicilin amyloids inhibit the fibrillogenesis of these proteins. In particular, vicilin amyloids decrease the number and length of lysozyme amyloid fibrils; the length and width of β-2-microglobulin fibrils; the number, length and the degree of clustering of β-amyloid fibrils; and, finally, they change the structure and decrease the length of insulin fibrils. Such drastic influences of vicilin amyloids on the pathological amyloids’ formation cause the alteration of their toxicity for mammalian cells, which decreases for all tested amyloids with the exception of insulin. Taken together, our study, for the first time, demonstrates the anti-amyloid effect of vicilin fibrils and suggests the mechanisms underlying this phenomenon.

## 1. Introduction

The formation of ordered fibrillar protein aggregates, amyloids, is associated with a wide range of disorders, such as Alzheimer’s and prion diseases, amyotrophic lateral sclerosis, various localized and systemic amyloidoses, etc. [1,2,3,4,5]. At the same time, recent studies indicate that proteins in the amyloid state can perform important biological functions [6,7] in different organisms: archaea, bacteria, fungi, plants, insects and mammals [8,9,10,11]. These functions include but are not limited to the mechanical protection and modification of the cell surface properties, biotic or abiotic surface adhesion, pigment biosynthesis, storage and release of hormones and toxins and participation in host–pathogen and host–symbiont interactions [12,13,14,15,16,17,18].

Interactions between different amyloid-forming proteins are supposed to affect the pathogenesis of different disorders like Alzheimer’s and Parkinson’s diseases [19,20,21] and are also important for the implementation of different biological functions like necroptosis signaling [22]. The co-aggregation of amyloids can modulate their physicochemical properties and can lead, in several cases, to the formation of hetero-amyloid aggregates consisting of different proteins [22,23]. The aggregation of a protein can be triggered by preformed amyloid oligomers called “seeds” of the same protein or by another protein (heterological cross-seeding). Such “seeds” can either be formed by endogenous protein molecules or, in particular cases, be exogenously transmitted [24]. The heterological cross-seeding is considered to play an important role in the development of different amyloidoses [25,26], and probably mediates several biological functions of amyloids [27,28].

One of the recently identified functional amyloids is the 7S seed storage globulin vicilin of the garden pea *Pisum sativum* L. This seed storage protein forms amyloids that accumulate during the seed maturation and disassemble after germination. The amyloids formed by vicilin in plant seeds are highly resistant to digestive enzymes under near physiological conditions and withstand food processing persisting in canned peas [10]. Therefore, we hypothesize that vicilin amyloids, which enter the digestive tract with peas or food prepared from them, could potentially interact with different human amyloid-forming proteins and modulate their aggregation.

In the present work, we tested the fundamental possibility of such an effect by analyzing the influence of vicilin amyloids preliminarily prepared in vitro on the formation, structure and cytotoxicity of fibrils formed from several mammalian proteins and peptides whose amyloid states are associated with the pathogenesis of Alzheimer’s disease; systemic lysozyme and hemodialysis amyloidoses; and localized insulin amyloidosis. We demonstrated that vicilin amyloid fibrils inhibit the formation of different human pathological amyloids, leading to a decrease in the number of fibrils, changing their morphology or structure. These data suggest that amyloids from plant seeds ingested with food could potentially inhibit the aggregation of human proteins, thereby modulating their pathological and functional effects.

## 2. Results

### 2.1. Amyloids Formed from Vicilin and Its Fragments Effectively Inhibit the Growth of Lysozyme Amyloid Fibrils

The lysozyme is a widely used model protein for the study of fibrillogenesis since its folding–unfolding processes, structure and stability are well known. Given the relatively high content of the lysozyme in saliva (about 0.1 mg/mL [29]) and mucosa of the gastrointestinal tract (GIT), as well as the fact that amino acid substitutions in a lysozyme can cause the accumulation of large amounts of amyloids in the liver, kidneys and other parts of the GIT [30,31], we chose the lysozyme as the first object of our study. We analyzed the effect on lysozyme fibrillogenesis not only of amyloids formed from full-length vicilin but also fibrils from its fragments cupin-1.1 and cupin-1.2 (Figure S1), whose amyloidogenic properties were proved by us earlier [10].

**Figure 1:**
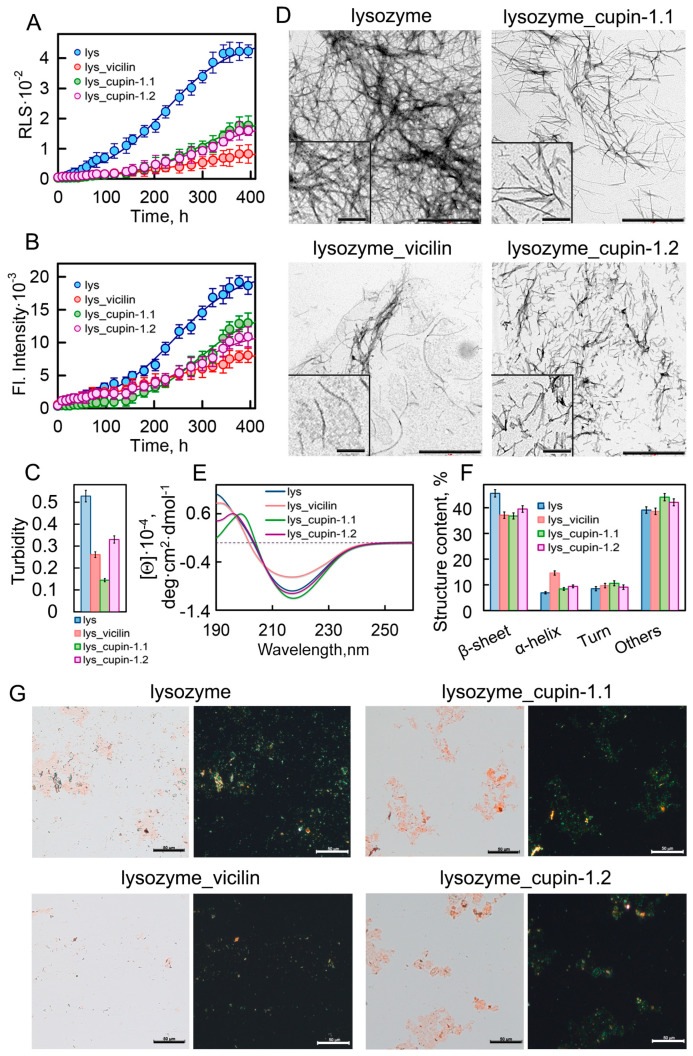
Inhibition of lysozyme fibrillogenesis in the presence of vicilin amyloids. (**A**) Rayleigh light scattering and (**B**) ThT fluorescence intensity characterizing the growth kinetics of lysozyme fibrils (1 mg/mL) in the absence (blue circles) and the presence of amyloids (0.05 mg/mL) formed from vicilin (red circles), cupin-1.1 (green circles) and cupin-1.2 (purple circles). (**C**) Turbidity of amyloids. The lines of best fit through the data points at (**B**,**C**) were obtained by fitting the data with a sigmoidal function. (**D**) TEM images of lysozyme fibrils formed in the absence and the presence of amyloids formed from vicilin, cupin-1.1 and cupin-1.2. The scale bars correspond to 1 μm. The insets show the zoomed-in image of amyloids. The scale bars are equal to 200 nm. (**E**) The far-UV CD spectra of the samples, and (**F**) CD spectra deconvolution using the CDPro and BeStSel methods. Here, we represent the change in the content of β-strands, α-helices, β-turns and other structures (including 3-10-helices, bends and unordered structures). Data are expressed as the mean ± SEM based on triplicate samples. The decoding of the colors used in (**C**,**E**,**F**) is the same as that in (**A**,**B**). (**G**) Birefringence of lysozyme fibrils formed in the absence and the presence of amyloids formed from vicilin, cupin-1.1 and cupin-1.2. Left—transmitted light, right—polarized light. Scale bar is equal to 50 μm. The concentration of the lysozyme fibrils at (**D**–**G**) is equal to 0.5 mg/mL.

We investigated the growth kinetics of lysozyme amyloid fibrils in the presence and absence of garden pea amyloids by recording the Rayleigh light scattering (RLS) of the forming aggregates (Figure 1A) and the fluorescence intensity of the amyloid-specific fluorescent probe thioflavin T (ThT) (Figure 1B). Calculated on the basis of these results, the values of the lag time (*τ_lag_*) of lysozyme fibrillogenesis in the presence of plant amyloids are similar or, in most cases, higher than in the control sample (Table S1). The apparent growth constant (*K_app_*) for the sample with vicilin amyloids is lower than in the control sample, indicating a deceleration in lysozyme fibril elongation. Significantly lower values of the recorded RLS and ThT fluorescence in the samples treated with amyloids formed from vicilin or its fragments compared to the control sample indicate the formation in them of a smaller number of lysozyme aggregates. The most pronounced decrease in these parameters was observed in the presence of full-size vicilin fibrils.

The inhibitory effect of vicilin amyloids could be due to their interaction: (1) with monomeric proteins, preventing the formation of amyloid seeds; (2) with formed amyloid seeds, preventing fibril elongation; and (3) with mature amyloid fibrils, preventing their interaction with each other and clustering. To identify the most probable of the assumptions made, we analyzed the interaction of vicilin amyloids with monomeric/aggregated forms of the lysozyme. To carry this out, we recorded the circular dichroism (CD) spectra of monomeric/aggregated forms of the lysozyme and their mixture with vicilin amyloids (Figure S2A–C). The concentration of vicilin fibrils was chosen so that they did not contribute to the recorded CD spectra. Thus, the interaction of various forms of the lysozyme with vicilin amyloids could be detected with the change in the CD spectrum of the samples after the addition of the latter (as it was made in ref. [32]).

The results obtained indicate the absence of changes in the CD spectra of a monomeric lysozyme in the presence of vicilin amyloids, which, however, cannot unequivocally reflect the absence of their interaction. To prove/disprove the formation of a complex between a monomeric lysozyme and vicilin amyloids, additional measurements of intrinsic lysozyme fluorescence (because of the presence of Trp residues in this protein structure) were conducted (Figure S2D). We performed a comparative analysis of a number of fluorescent characteristics of a lysozyme in the absence and presence of vicilin amyloids, which may indicate conformational rearrangements and changes in the spatial structure of the protein (Table S2): integrated fluorescence intensity (*F_total_*), the wavelength of the fluorescence spectrum maximum (λ*_max_*), parameter *A* (which is the ratio of fluorescence intensities of the sample at two wavelengths—320 and 365 nm—and is sensitive to changes in the position and shape of fluorescence spectra [33,34]) and fluorescence anisotropy (*r*). The equality of the determined parameters (Table S2) indicates a similarity of polarity and rigidity of the microenvironment of tryptophan residues responsible for lysozyme fluorescence, as well as the same mobility of protein tryptophan residues in the tested samples. This is in good agreement with the CD spectroscopy data (Figure S2A–C) about the lowest probability of the interaction of a vicilin amyloid with a monomeric native lysozyme. This may be because of its compact globular structure, with the amyloidogenic regions located inside the protein globule. However, we observed noticeable changes in the CD spectra during lysozyme fibrillogenesis and after its completion, which indicates the possibility of the interaction of vicilin amyloids with intermediate forms of the lysozyme/amyloid seeds/mature amyloids. We believe that this interaction leads to the observed inhibitory effect of vicilin amyloids.

This effect is also confirmed with the decrease in the turbidity of the studied samples (Figure 1C) and with their visualization using polarization microscopy in the presence of Congo Red dye (Figure 1G). The visualization of amyloids using transmission electron microscopy (TEM) showed that the lysozyme amyloids in the control sample were long thin fibrils collected in bundles (Figure 1D). Despite the fact that the TEM method is more suitable for qualitative estimates, we nevertheless attempted to calculate the linear sizes of the fibrils in samples using images taken in different fields of view of the microscope. The results obtained indicate that the presence of garden pea protein amyloids in the sample during fibrillogenesis led to a significant decrease not only in the number but also in the length of lysozyme fibrils (Figure S3).

To characterize the structure of fibrils formed from the lysozyme in the absence and presence of the vicilin, cupin-1.1 and cupin-1.2 amyloids, we recorded the CD spectra of the samples in the far UV region (Figure 1E). The analysis of the spectra using BeStSel (https://bestsel.elte.hu/index.php, accessed on 1 January 2023) and CDpro (https://sites.google.com/view/sreerama, accessed on 1 January 2023) software packages led to the conclusion that the presence of garden pea amyloids leads to a decrease in the content of β-sheets in the sample, which form the backbone of the lysozyme fibril (Figure 1F). This not only confirms a decrease in the number of formed amyloid fibrils but may also indicate a change in their structure.

To test this assumption, we analyzed the interaction of fibrils with fluorescent probe ThT, which, according to the generally accepted model, binds to the β-sheets backbone of amyloids along the long axis of the fibril perpendicular to the β-sheets [35] and can be used to analyze their structural polymorphism (see, for example, [36,37,38,39]). To characterize the photophysical properties of the fibril-bound dye, we performed a spectroscopic study of ThT samples with amyloids prepared using equilibrium microdialysis, followed by the correction of the recorded fluorescence intensity values for the primary inner filter effect [40]. It turned out that the values of the fluorescence quantum yield and lifetime of the dye bound to lysozyme fibrils, obtained in the presence and the absence of vicilin amyloids, are the same (Table 1). This indicates the identity of the microenvironment of ThT molecules bound to amyloids, and, consequently, the structure of the studied amyloid fibrils.

The results obtained indicate that the presence of amyloids formed from vicilin and its fragments does not lead to a change in the structure, but at the same time, noticeably reduces the number and length of lysozyme amyloid fibrils, and, therefore, inhibits their growth. Since we observed the most pronounced inhibitory effect on lysozyme fibrillogenesis in the presence of full-length vicilin amyloids, further studies were carried out using vicilin fibrils.

### 2.2. Vicilin Amyloids Cause a Decrease in the Length of β-2-Microglobulin Fibrils

To find out whether the effects of vicilin fibrils on the growth of other amyloids are more general, we investigated the impact of vicilin amyloids on the β-2-microglobulin (β2m) amyloids’ formation. The β2m amyloids form in vivo during the long-term persistence of high protein concentrations in the body of patients with acute renal failure who are on long-term hemodialysis treatment [41]. The β2m amyloidosis can manifest in the form of carpal tunnel syndrome, periarthrosis, arthropathy, bursitis, bone cysts, pathological fractures, etc. [42,43,44,45,46,47,48].

**Figure 2:**
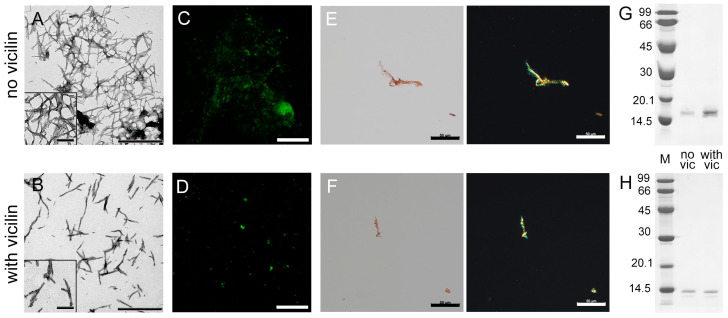
Reducing the length of β2m fibrils under the action of vicilin amyloids. TEM images of β2m fibrils (0.5 mg/mL) formed (**A**) in the absence and (**B**) in the presence of vicilin amyloids (0.025 mg/mL). The scale bars correspond to 1 μm. The insets of (**A**,**B**) show the zoomed-in image of amyloids. The scale bars are equal to 200 nm. Fluorescence images of the stained-by-ThT β2m fibrils formed (**C**) in the absence and (**D**) in the presence of vicilin amyloids. The scale bar is equal to 10 μm. Birefringence of β2m fibrils formed (**E**) in the absence and (**F**) in the presence of amyloids formed from vicilin. Left—transmitted light, right—polarized light. Scale bars are equal to 50 μm. Pseudo-native SDS-PAGE of the samples with (**G**) lysozyme and (**H**) β2m fibrils formed in the absence (no vic) and the presence (with vic) of vicilin amyloids. Lysozyme and β2m amyloids were loaded on the gel at 0.25 mg/mL concentration. “M” corresponds to marker proteins. Molecular weights (kDa) are shown.

We investigated β2m fibrillogenesis in the absence and presence of vicilin amyloids in vitro. Samples with β2m aggregates were visualized using transmission electron (Figure 2A,B), confocal laser scanning (Figure 2C,D) and polarized microscopy (Figure 2E,F). It turned out that, as in the case of the lysozyme, shorter and thinner β2m fibrils were formed in the sample incubated in the presence of vicilin amyloids compared to the control sample (Figure S3). At the same time, vicilin amyloids led only to a slight decrease in the turbidity and RLS of β2M fibrils, as well as the fluorescence intensity of bound-to-amyloids ThT (Table 1). This probably indicates that the effect of fibril shortening on these characteristics is offset by a simultaneous increase in their number in the sample. Thus, apparently, the presence of vicilin amyloids does not prevent the formation of β2m fibrils (in contrast to the case of the lysozyme) but only reduces their length. This assumption is in good agreement with the data of CD spectroscopy, which indicate the predominant binding of vicilin amyloids to the fibrillar, but not monomeric, form of β2m (Figure S2E,F). Probably, such interaction prevents the elongation of the already formed β2m fibrils.

Our assumption is also confirmed with the data of pseudo-native SDS-PAGE, which allowed for estimating the proportion of monomeric fractions in the samples. High-molecular-weight amyloids resistant to cold SDS treatment did not enter in the gel, while monomers migrated as bands of different molecular weights. It was shown that the band corresponding to a monomeric lysozyme in the sample incubated in the presence of vicilin amyloids is noticeably more intense than in the control (Figure 2G). For the quantitative assessment of changes in the monomeric fraction content in the samples, the bands on the SDS-PAGE were analyzed using ImageJ 1.53k software. Data were normalized to the area of the bands in the control samples (Figure S4). The results confirm that the presence of vicilin amyloids prevents the transition of a monomeric lysozyme to the fibrillar form. In the case of β2m, this effect does not appear: the content of the monomeric fraction after the completion of fibrillogenesis is almost the same (Figure 2H and Figure S4). The results obtained correspond to the same content of β-sheets (forming the fibril backbone) in the β2m samples (Table 1). The latter, along with the data on the identity of the photophysical characteristics of ThT bound to these amyloids (Table 1), also shows no difference in the structure of β2m fibrils formed in the presence or absence of vicilin amyloids.

Thus, it was shown that vicilin amyloids do not completely prevent β2m amyloidogenesis; however, they significantly reduce β2m fibril length.

### 2.3. Vicilin Amyloids Inhibit the β-Amyloid Fibril Clustering

**Figure 3:**
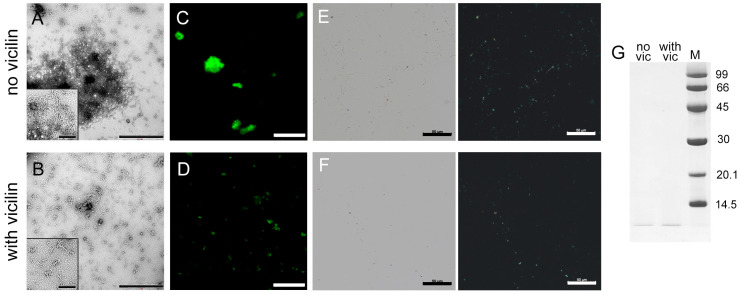
Inhibition of Aβ42 fibrils clustering under the action of vicilin amyloids. TEM images of Aβ42 fibrils (0.5 mg/mL) formed (**A**) in the absence and (**B**) in the presence of vicilin amyloids (0.025 mg/mL). The scale bars correspond to 1 μm. The insets of (**A**,**B**) show the zoomed-in image of amyloids. The scale bars are equal to 200 nm. Fluorescence images of the stained-by-ThT Aβ42 fibrils formed (**C**) in the absence and (**D**) in the presence of vicilin amyloids. The scale bar is equal to 10 μm. Birefringence of Aβ42 fibrils formed (**E**) in the absence and (**F**) in the presence of amyloids formed from vicilin. Left—transmitted light, right—polarized light. Scale bar is equal to 50 μm. (**G**) Pseudo-native SDS-PAGE of the sample with Aβ42 fibrils formed in the absence (no vic) and the presence (with vic) of vicilin amyloids. Aβ42 amyloids were loaded on the gel at 0.25 mg/mL concentration. “M” corresponds to marker proteins. Molecular weights (kDa) are shown.

The next object of study was the 42-residue β-amyloid peptide (Aβ42), which forms amyloid plaques in the brains of patients with Alzheimer’s disease [49,50]. TEM data suggest that, in vitro, Aβ42 forms amyloids, which are thin fibrils that interact with each other and form large clusters (Figure 3A). Although most of the fibrils in the control sample form clusters, we tried to estimate the length of the fibrils outside the clots. The length of the fibrils obtained in the presence of vicilin is shorter than in the control sample (Figure S3). It should be noted that Aβ42 fibrils formed in the presence of vicilin amyloids, in contrast to the fibrils in the control sample, are separate thin, short and curved fibrils with a significantly lower tendency to form clusters (Figure 3B). The lesser tendency to the clumping of Aβ42 fibrils formed in the presence of vicilin amyloids is also confirmed with confocal laser scanning microscopy data (Figure 3C,D). It can be assumed that the interaction of Aβ42 fibrils with each other is inhibited by their binding to vicilin amyloids that creates steric or charge restrictions for additional intramolecular contacts. This assumption agrees with the CD spectroscopy data, which confirm the binding of vicilin amyloids to the fibrillar form of Aβ42 (Figure S2G). At the same time, we noted that the photophysical characteristics of ThT bound to fibrils formed in the absence and the presence of vicilin amyloids were the same (Table 1), which indicates the identity of the structure of Aβ42 amyloid fibrils.

The reduction in the turbidity and RLS of Aβ42 aggregates in the sample with vicilin amyloids compared to the control sample (Table 1) is in good agreement with the assumption of a decrease in the size of amyloid clusters in the sample. However, such a decrease may also indicate a decrease in the total number of amyloid fibrils due to the partial inhibition of their formation. To test this assumption, we compared the fluorescence intensity of fibril-bound ThT, the content of the β-sheet structure (Table 1) and the amount of aggregated (Figure 3E,F) and monomeric forms of the peptide (Figure 3G and Figure S4) in the samples. Taking into account the experimental error, a slight decrease in the intensity of ThT fluorescence and the number of β-sheets, as well as a visual decrease in the length and amount of aggregates and an increase in the intensity of the band corresponding to the monomeric peptide in the sample with vicilin amyloids compared to the control, indicate that the inhibition of the growth of Aβ42 fibrils takes place; however, the effect found is less significant compared to the effect found in the case of the lysozyme.

Thus, it was concluded that vicilin amyloids prevent the interaction of Aβ42 fibrils with each other and their clustering, and, to a small extent, reduce their number.

### 2.4. Vicilin Amyloids Alter the Structure of Insulin Fibrils

Another object of study in this work was insulin protein, which, like the lysozyme, is considered a convenient model object for studying fibrillogenesis. In addition, the number of works aimed at the study of the local iatrogenic amyloidosis caused by insulin aggregation is increasing [51,52,53,54,55,56,57,58]. Insulin amyloid fibrils were found in patients with type II diabetes mellitus after the repeated or even single subcutaneous injection of insulin. Injected insulin can form fibrils in the body of patients regardless of the injection site, for example, in the thighs [52,53], shoulders [55], arms [56] and abdominal wall [55,56,57].

**Figure 4:**
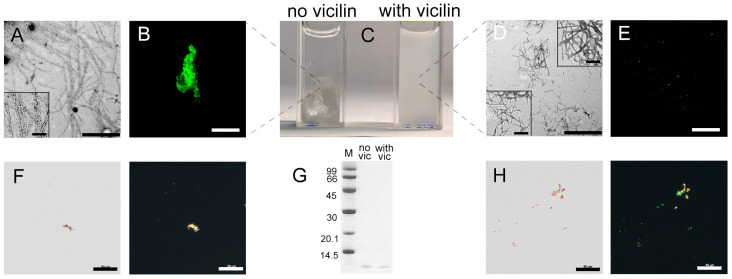
Change in insulin amyloid fibrils induced with vicilin amyloids. TEM images of insulin fibrils (0.5 mg/mL) formed (**A**) in the absence and (**D**) in the presence of vicilin amyloids (0.025 mg/mL). The scale bars correspond to 1 μm. The bottom insets of (**A**,**D**) show the zoomed-in image of insulin amyloids. The top inset of (**D**) shows the zoomed-in image of vicilin amyloids. The scale bars are equal to 200 nm. (**C**) Visualization of the insulin fibrils’ suspensions (1 mg/mL). (**B**,**E**) Fluorescence images of the stained-by-ThT insulin fibrils formed (**B**) in the absence and (**E**) in the presence of vicilin amyloids. The scale bars are equal to 10 μm. (**F**) Birefringence of insulin fibrils formed in the absence of amyloids formed from vicilin. Left—transmitted light, right—polarized light. Scale bars are equal to 50 μm. (**G**) Pseudo-native SDS-PAGE of the sample with insulin fibrils formed in the absence (no vic) and the presence (with vic) of vicilin amyloids. Insulin amyloids were loaded on the gel at 0.25 mg/mL concentration. “M” corresponds to marker proteins. Molecular weights (kDa) are shown. (**H**) Birefringence of insulin fibrils formed in the presence of amyloids formed from vicilin. Left—transmitted light, right—polarized light. Scale bars are equal to 50 μm. The concentration of the insulin fibrils at (**B**,**E**,**F**,**H**) is equal to 0.5 mg/mL.

TEM data indicate that the amyloid fibrils formed from insulin in vitro have the greatest thickness and length compared to all amyloids studied by us in this work (Figure 4A), and form large aggregates (Figure 4B,F) seen as a pronounced precipitate when the sample is incubated without stirring (Figure 4C). However, the addition of vicilin amyloids during insulin fibrillogenesis leads to a noticeable decrease in the size of the mature fibrils (Figure 4D and Figure S3) and their aggregates (Figure 4E). This is in good agreement with the reduction in the turbidity and RLS of insulin fibrils incubated with vicilin amyloids (Table 1), as well as the absence of a pronounced precipitate in the sample (Figure 4C). At the same time, the amount of the aggregated (Figure 4F,G) and monomeric form of the protein in this sample does not increase (Figure 4H and Figure S4), which indicates that vicilin amyloids do not prevent the formation of insulin fibrils. Interestingly, in the case of insulin in contrast with other proteins, we observed a slight change in the CD spectrum of the monomeric protein, although the interaction with the fibrillar insulin form is more obvious (Figure S2H,I). Nevertheless, these data allow for assuming some influence of vicilin amyloids on the early stages of insulin fibrillogenesis. 

Even though the amount of monomeric insulin did not increase after the addition of vicilin amyloids, we observed a decrease in the content of β-sheets in the sample (Table 1). It is also important to note that, unlike other amyloid fibrils, in the case of insulin fibrils, the presence of vicilin amyloids resulted not in a decrease, but in a marked increase in the intensity of ThT fluorescence (Table 1), as well as in a decrease in the diameters and lengths of amyloid fibrils (Figure 4D). In addition, the photophysical characteristics of ThT bound to insulin fibrils prepared in the presence and in the absence of vicilin amyloids are different (Table 1).

Taken together, these data indicate a change in the structure and morphology and a decrease in linear dimensions of insulin fibrils during their preparation in the presence of vicilin amyloids.

### 2.5. The Effect of Vicilin Amyloids on the Fibrillogenesis Depends on the Duration of the Lag Phase

We demonstrated that vicilin amyloids affect the fibrillogenesis of all tested mammalian amyloids. Nevertheless, the influence of vicilin was the most pronounced for the lysozyme. We hypothesized that this effect may be explained with the fact that the lag phase of lysozyme fibrillogenesis under the selected conditions is the longest in comparison with β2m and Aβ42 (several days), which could increase the probability of interaction between a vicilin amyloid and lysozyme monomer, resulting in the strong inhibition of the lysozyme fibril formation.

**Figure 5:**
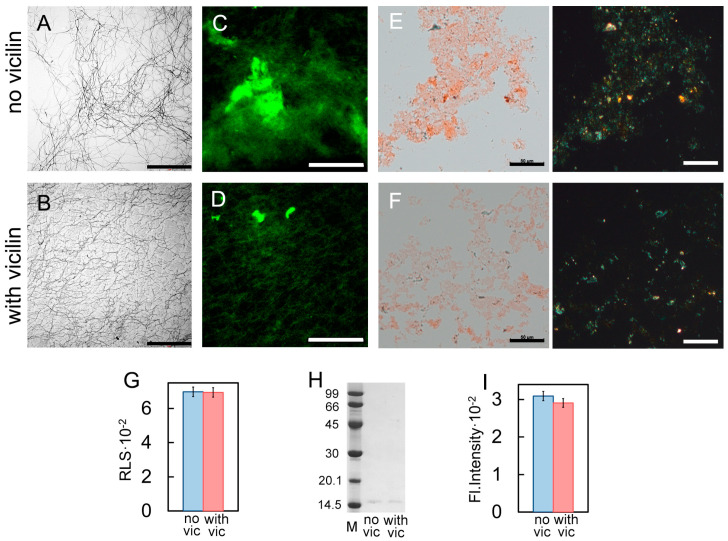
Change in the effect of vicilin amyloids on lysozyme fibrils with an increase in the rate of their fibrillogenesis. TEM images of lysozyme fibrils (0.5 mg/mL) formed (**A**) in the absence and (**B**) in the presence of vicilin amyloids (0.025 mg/mL). The scale bars correspond to 2 μm. Fluorescence images of the stained-by-ThT lysozyme fibrils formed (**C**) in the absence and (**D**) in the presence of vicilin amyloids. The scale bar is equal to 10 μm. Birefringence of lysozyme fibrils formed (**E**) in the absence and (**F**) in the presence of amyloids formed from vicilin. Left—transmitted light, right—polarized light. Scale bar is equal to 50 μm. (**G**) Rayleigh light scattering and (**H**) ThT fluorescence intensity of lysozyme fibrils formed in the absence (no vic) and the presence (with vic) of vicilin amyloids. The concentration of the lysozyme fibrils at (**A**–**G**) and (**I**) is equal to 0.5 mg/mL. (**I**) Pseudo-native SDS-PAGE of the sample with insulin fibrils formed in the absence (no vic) and the presence (with vic) of vicilin amyloids. Lysozyme amyloids were loaded on the gel at 0.25 mg/mL concentration. “M” corresponds to marker proteins. Molecular weights (kDa) are shown.

To confirm the proposed hypothesis, we accelerated the fibrillogenesis of the lysozyme using the additional mixing of the sample, which led to a significant decrease in the duration of the lag phase (it was less than a day). The analysis of the obtained samples with transmission electron (Figure 5A,B), confocal laser scanning (Figure 5C,D) and polarized microscopy (Figure 5E,F), as well as the registration of their RLS (Figure 5G), the ThT fluorescence intensity (Figure 5I) and the assessment of the content of the monomer fraction in the samples (Figure 5H and Figure S4), made it possible to conclude that the presence of vicilin amyloids did not have a pronounced inhibitory effect on lysozyme fibrillogenesis in these conditions. As in the case of β2m and Aβ42 amyloids, we observed only a length and width reduction in the formed fibrils (Figure S3) without a noticeable decrease in their number (Figure 5A–F). Thus, we confirmed the assumption that the duration of the fibrillogenesis lag phase plays an important role in the process of heterological fibrillogenesis inhibition with vicilin amyloids.

### 2.6. Vicilin Amyloids Reduce the Cytotoxicity of Lysozyme, β2m and Aβ42 Fibrils and Increase the Cytotoxicity of Insulin Amyloids

**Figure 6:**
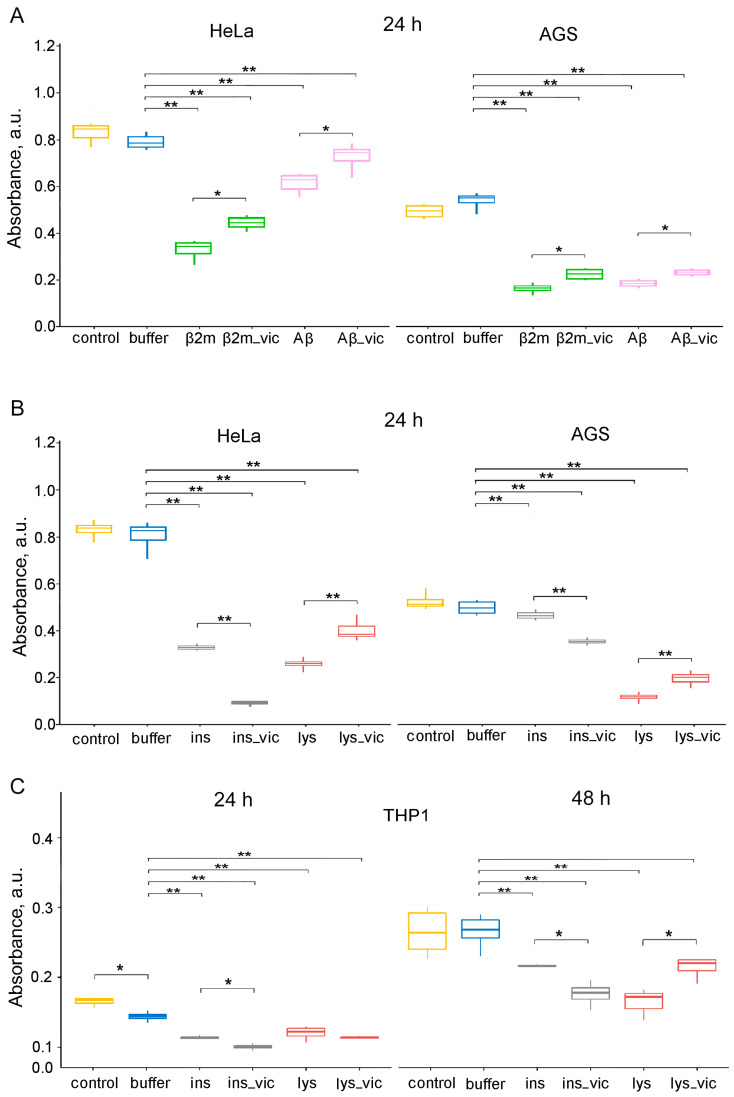
The data of MTT assay for evaluation of the metabolic activity of HeLa, AGS and THP-1 cell lines. Cells were exposed to (**A**) β2m or Aβ42 (Aβ), (**B**,**C**) lysozyme (lys) or insulin (ins) amyloids prepared in the absence and the presence of vicilin amyloids (lys_vic, etc.) in concentration of about 0.01 mg/mL for 24 (**A**–**C**) and 48 (**C**) hours. Data are given as the minimum and maximum, the sample median and the interquartile range for triplicate samples (HeLa and AGS), and as the mean ± SEM for four replicates (THP-1). * *p* ≤ 0.05, ** *p* ≤ 0.01.

Another intriguing issue that we tried to resolve in this work was the effect of vicilin amyloids on the cytotoxicity of fibrils formed from various mammalian amyloidogenic proteins. We analyzed the cytotoxic properties of the lysozyme, β2m, Aβ42 and insulin fibrils, formed in the absence or presence of vicilin amyloids, on such a widely used factor for this kind of research and highly sensitive to an amyloid model object as human cervical cancer (Hela) using the MTT test (Figure 6A,B). First, we showed that vicilin amyloids at a 5% (*v*/*v*) concentration (in which they were added to samples with mammalian proteins) did not affect cell viability (Figure S5). At the same time, vicilin amyloids at a higher concentration (equal to the concentration of mammalian proteins in the samples) turned out to be toxic to Hela cells (Figure S5). This is in good agreement with the data on the toxicity of these amyloids to mammalian cells obtained in the work [10].

It was shown that the cytotoxicity of the lysozyme, β2m and Aβ42 fibrils formed in the presence of 5% vicilin amyloids was lower than in the case of control samples. The most pronounced increase in cell viability after exposure to vicilin amyloids was observed for lysozyme fibrils (more than 1.5 times). At the same time, insulin fibrils whose structure was changed under the influence of vicilin amyloids (Figure 4), on the contrary, increased their cytotoxicity in comparison with the control sample (by 3.7 times) (Figure 6B). We tested the versality of the detected effects on another model cell line, human gastric adenocarcinoma (AGS) cells (Figure 6A,B). The change in the effect of amyloids obtained in the presence of vicilin amyloids on both tested cell lines turned out to be identical; however, the severity of these changes was different.

Similar results have been shown for the human monocytic leukemia cell line THP-1 (ATCC TIB-202). Assessing its viability with the MTT test after incubation with lysozyme and insulin fibrils formed in the presence or absence of vicilin amyloids shows an increase in insulin amyloids’ toxicity in the presence of vicilin (Figure 6C). The effect of vicilin amyloids’ presence on the properties of lysozyme fibrils is opposite; the presence of vicilin reduces lysozyme fibrils’ toxicity. Interestingly, the effect was significant only after 48 h of incubation and was not seen after 24 h, unlike with insulin. The difference in the toxicity of insulin fibrils with or without vicilin amyloids was significant after 24 h of incubation with cells (Figure 6C).

Thus, we showed that vicilin amyloids significantly affect the process of mammalian amyloids’ formation by altering their structure, size and other properties, resulting in changes in their toxicity for mammalian cells.

## 3. Discussion

In the present work, it was shown, for the first time, that amyloids formed from the *P. sativum* L. seed storage protein vicilin cause a general inhibitory effect on the amyloid fibril formation, but the features of this effect significantly vary regarding different amyloids. In particular, we demonstrated the following: (1) a decrease in the number and length for lysozyme amyloid fibrils, (2) decrease in the length and width of β2m fibrils, (3) decrease in the length, degree of clustering and number of Aβ42 amyloid fibrils and (4) change in the structure and decrease in the length of insulin fibrils. It should be noted that these amyloid deposits, in some cases, were detected directly in the gastrointestinal tract or adjacent tissues, which indicates the potential possibility of their interaction with vicilin amyloids in vivo [59,60,61,62,63].

**Figure 7:**
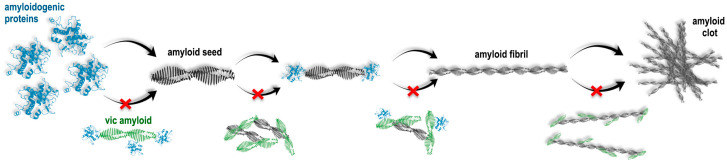
Schematic presentation of suggested pathways for the action of vicilin amyloids on the formation of fibrils from mammalian amyloidogenic proteins. It is shown that vicilin amyloids could interact (1) with monomeric proteins, preventing the formation of amyloid seeds, (2) with formed amyloid seeds, preventing fibril elongation and (3) with mature amyloid fibrils, preventing their interaction with each other and clustering. Red cross symbols show what structural transitions can be inhibited due to the action of vicilin amyloids.

It can be assumed that the observed inhibition of fibrillogenesis could occur in the case of the interaction of vicilin amyloids with monomeric amyloidogenic proteins, which, in turn, leads to the impossibility of their binding to each other and the formation of an amyloid fibril (Figure 7). We could suppose that vicilin amyloids either affect (1) amyloidogenic regions of mammalian proteins or areas in their immediate vicinity, sterically preventing fibrillogenesis, or (2) non-amyloidogenic regions, changing the properties of the surface of mammalian proteins exposed to a solvent, thus preventing these proteins from binding to each other. In this regard, the following question arises: why is the prevention of fibril growth with vicilin amyloids the most pronounced in the case of the lysozyme? To answer this question, first of all, it should be noted that the process of amyloid formation can be divided into three main stages: the lag phase (the process of nucleation), the phase of exponential growth (fibril elongation) and the saturation phase (the assembly of protofibrils into mature multistranded amyloid fibrils with different morphologies) [64,65,66]. We proposed that a relatively long duration of the lag phase of amyloid formation with the lysozyme (Figure 1A,B) may increase the probability of the interaction of this protein in an intermediate state with vicilin amyloids, resulting in the termination of lysozyme fibril formation. The experiments on shortening the lysozyme fibril formation lag phase with additional mixing confirmed this hypothesis (Figure 5). Thus, the lag phase duration is a crucial factor for the vicilin amyloid interactions with other amyloidogenic proteins.

According to our hypothesis, due to a relatively short lag phase, the monomers of β2m, Aβ42 and the lysozyme (in the presence of mixing) do not have sufficient time to interact with vicilin amyloids and begin to form amyloid fibrils. However, in this case, vicilin amyloids can interact with already formed amyloid oligomers, preventing their further elongation or interaction with each other (Figure 7). This is in good agreement with a decrease in the length of β2m and lysozyme fibrils and the degree of Aβ42 fibril clustering (Figure 1, Figure 2 and Figure 3).

Considering the longest duration of insulin fibrillogenesis (about 2 weeks), it can be assumed that vicilin aggregates also effectively interact with its monomers, as with lysozyme monomers. However, we do not observe a decrease in the number of fibrils, but we observe a change in their structure and diameter. It can be assumed that vicilin fibrils interacting with monomeric insulin do not inhibit their aggregation, but, on the contrary, act as “seeds” for the amyloid’s formation from target protein (in our case, insulin) with altered morphology. However, there are two factors that do not allow us to confirm the assumption about seeding: (i) an almost two-fold difference in the fluorescence quantum yields of fibril-bound ThT (Table 1), and (ii) no acceleration of insulin fibrillogenesis in the presence of vicilin amyloids. Another possible interpretation of the results obtained is only partial shielding of the amyloidogenic insulin fragment due to interaction with vicilin amyloids, which leads to the formation of an insulin amyloid fibril from the remaining unshielded amyloidogenic region (smaller than in the case of intact amyloids). This assumption agrees with the fact that insulin fibrils, after exposure to vicilin amyloids, have a lower content of β-sheets (Figure 4, Table 1) that form the fibril backbone.

An important and unexpected result of this study is that different mechanisms underlying the inhibitory effects of vicilin amyloids on the fibril formation with various mammalian proteins can cause both an increase and a decrease in the cytotoxicity of these fibrils (Figure 6). In particular, we demonstrated that a decrease in the number, length or degree of clustering of amyloid fibrils (lysozyme, β2m and Aβ42) under the action of vicilin amyloids leads to a decrease in their toxic effects on cells, while a change in the structure of mature fibrils (insulin), on the contrary, can lead to an increase in cytotoxicity. Such structural alterations because of co-aggregation are known to increase the toxicity of amyloids, as in the case of an islet amyloid polypeptide (IAPP) and Aβ [67]. Similar effects were shown by us earlier when changing the conditions of fibrillogenesis and the amino acid sequence of the studied mammalian proteins and peptides, as well as when exposing their amyloids to proteins with chaperone and protease activity [39,68,69].

Cross-inhibitory interactions are described for several amyloids. For example, transthyretin inhibits Aβ amyloid formation by suppressing its nucleation and leading to the formation of non-amyloid aggregates [70,71]. Also, transthyretin impairs the lag phase of IAPP amyloid formation, causing fibrillogenesis inhibition [72]. Notably, a decrease in the toxicity was observed by us in the cases of three out of four (Aβ42, β2m and lysozyme) amyloids formed in the presence of vicilin fibrils, and only for insulin fibrils was an increase in toxicity caused by the structural alterations registered. Interestingly, vicilin amyloids are toxic for mammalian cells in vitro [10] but their influence reduces the toxicity of Aβ42, β2m and lysozyme amyloids in similar conditions. The fact that functional plant amyloids can inhibit the fibrillogenesis of mammalian amyloids may be important for the future development of approaches to the prevention and treatment of amyloidoses. However, it should be noted that in addition to pathological amyloids, humans also have functional ones [8,11,15], whose interaction with vicilin was not studied in this work. Taking into account the general inhibitory effect of vicilin amyloids on various pathological amyloids, one could assume a similar effect of vicilin amyloids on functional human amyloids, the modulation of aggregation of which could potentially have adverse consequences. For this reason, the issue of using plant amyloid proteins as inhibitors of amyloidogenesis needs to be clarified in future studies.

Overall, in this study and for the first time, we demonstrated that functional plant amyloid vicilin inhibits the aggregation of pathological mammalian amyloids via decreasing fibril growth or modulating their structure and morphology, thus altering the toxicity of these amyloids for mammalian cells.

## 4. Materials and Methods

### 4.1. Materials

Guanidine hydrochloride (GdnHCl), the lysozyme, fluorescent dye thioflavin T (ThT) “UltraPure Grade” (AnaSpec, Fremont, CA, USA), buffer components, 3-(4,5-dimethylthiazol-2-yl)-2,5-diphenyltetrazolium bromide (MTT), 1,1,1,3,3,3-Hexafluoro-2-propanol (HFIP) were from Sigma (St. Louis, MO, USA); Aβ42 peptides were from (GL Biochem, Shanghai, China); DMEM (glucose, 4.5 g/L), fetal bovine serum (FBS) and 0.25% Trypsin-EDTA were from Gibco (Thermo Fisher Scientific, Waltham, MA, USA); Culture flasks and 96-well plates (flat bottom) were from Corning (Corning, NY, USA); a Dulbecco’s Modified Eagle Medium (DMEM), fetal bovine serum (FBS), penicillin and streptomycin from Gibco BRL (Life Technologies, Paisley, Scotland) were used without additional purification. culture flasks and 96-well plates (flat bottom) were purchased from Corning (USA). The samples of recombinant β2m were received from Mikhail M. Shavlovsky, Dmitry S. Polyakov and Rodion G. Sakhabeev (Department of Molecular Genetics, Institute of Experimental Medicine, Saint Petersburg, Russia).

### 4.2. Recombinant Protein Production and Purification

For vicilin, cupin-1.1 and cupin-1.2 expression, *E. coli* strain BL21 (New England Biolabs, Ipswich, MA, USA) and previously constructed pAc-Vicilin, pAc-Cupin-1.1 and pAc-Cupin-1.2 plasmids [10] were used. The overproduction of recombinant proteins was carried out in 2TYa media supplemented with 0.1 mM of IPTG. Cultures were grown at 37 °C for 4 h. Proteins were purified in denaturing conditions (in the presence of 8 M urea) according to a previously published protocol [73] without the Q-sepharose purification step. A one-step purification procedure with a Ni-NTA agarose (Invitrogen, Carlsbad, CA, USA) column was performed according to the manufacturer’s recommendations. Proteins were concentrated using ethanol instead of methanol used in the original protocol.

### 4.3. Amyloid Fibrils’ Preparation

Vicilin, cupin-1.1, cupin-1.2 and Aβ42 were dissolved in 50%-organic-solvent 1,1,1,3,3,3-Hexafluoro-2-propanol (HFIP) and incubated for 7 days [10,39,74,75]. The concentration of the proteins and peptide was 1 mg/mL (or 21, 59, 54 and 222 μM, respectively). Afterward, the HFIP was slowly evaporated under a stream of nitrogen, then the volume of the sample was adjusted with distilled water to the initial one, and the samples were stirred for an additional 7 days at 37 °C.

For the preparation of amyloid fibrils from β-2-microglobulin (β2m), the protocol described in work [38] was used. Protein in a concentration of 1 mg/mL (80 μkM) was incubated in the Gly-HCl buffer (pH 2.5) at 37 °C.

Lysozyme amyloid fibrils were prepared with the protein dissolving in 20% acetic acid/100 mM of NaCl (pH 2) at 37 °C [37] with and without stirring. Insulin amyloid fibrils were prepared with the protein dissolving in MQ water at 37 °C without stirring. The concentration of the protein was 1 mg/mL (70 μM).

Vicilin, cupin-1.1 and cupin-1.2 amyloids from stock solutions (1 mg/mL, or 21, 59 and 54 μM, respectively) after sonication (for 5 min at 37 °C in the water-bath-type ultrasonic transmitter Elmasonic P30H with a frequency of 37 kHz) were added to the samples with different proteins at the beginning of fibrillogenesis in a 5% (*v*/*v*) concentration. The constant temperature during fibril growth was maintained with a TS-100 Thermo-Shaker (Bio-san, Riga, Latvia).

Samples were diluted 2-fold for characterization using various physicochemical methods. To prove the direct interaction of mammalian proteins in monomeric and aggregated forms with vicilin amyloids using CD spectroscopy, a 0.5 mg/mL concentration of mammalian proteins and 0.1 mg/mL concentration of vicilin fibrils were used.

### 4.4. Transmission Electron Microscopy

Micrographs were obtained using a transmission electron microscope, Libra 120 (Carl Zeiss, Jena, Germany). The samples were placed on copper grids coated with formvar/carbon films (Electron Microscopy Sciences, Hatfield, PA, USA). To obtain electron micrographs, the method of negative staining with a 1% aqueous solution of uranyl acetate was used.

### 4.5. ThT–Amyloid Fibril Sample Preparation

ThT-fibrils-tested solutions were prepared with equilibrium microdialysis [40] using a Harvard Apparatus/Amika device (Holliston, MA, USA). This device consists of two chambers with an equal volume that are separated by a membrane impermeable to particles greater than 10 kDa (i.e., permeable to fluorescent probes and impermeable to amyloids). ThT at an initial concentration of 64 μM was placed in one of these chambers. Mature amyloid fibrils formed with different proteins in a concentration of 0.5 mg/mL were placed in chamber 2. After equilibrium, the dye concentrations in two chambers became equal (*C_f_*). The total dye concentration in the chamber with amyloids exceeded that in another chamber using the concentration of the bound dye (*C_b_* = *C*_0_ − 2*C_f_*). The spectroscopic study of the sample and reference solutions prepared with the proposed approach allowed us to determine the photophysical characteristics of ThT bound to tested amyloids.

### 4.6. Spectral Measurements

The absorption spectra of the samples were recorded using a U-3900H spectrophotometer (Hitachi, Tokyo, Japan). The absorption spectra of amyloid fibrils and ThT in the presence of the fibrils were analyzed along with the light scattering using a standard procedure [76]. The concentration of the samples was determined using the following molar extinction coefficients: ε_280_ = 14,900 (vicilin), ε_280_ = 8940 (cupin-1.1), ε_280_ = 4470 (cupin-1.2), ε_280_ = 1490 M^−1^cm^−1^ (Aβ42), ε_280_ = 36,000 M^−1^cm^−1^ (lysozyme), ε_276_ = 20,065 M^−1^cm^−1^ (β2m), ε_280_ = 5800 M^−1^cm^−1^ (insulin) and ε_412_ = 31,600 M^−1^cm^−1^ (ThT). The turbidity was recorded at 530 nm.

CD spectra in the far UV region were measured using a J-810 spectropolarimeter (Jasco, Tokyo, Japan). The CD spectrum of the appropriate buffer was recorded and subtracted from the sample’s spectra. The secondary structure content of amyloid samples was estimated with the BeStSel webserver [77], as it predicted well the secondary structure of amyloid aggregates enriched by β-sheets [78].

Fluorescence spectra of ThT (λ_ex_ = 440 nm and λ_em_ = 450–700 nm) and the lysozyme (λ_ex_ = 297 nm and λ_em_ = 300–600 nm) were measured using a Cary Eclipse spectrofluorimeter (Varian, Belrose, Australia). Recorded ThT fluorescence intensity was corrected on the primary inner filter effect with the use of a previously elaborated approach [79]. Rayleigh light scattering (RLS) was recorded at 530 nm (λ_ex_ = 530 nm). The kinetic parameters, apparent rate constant (*K_app_*) and lag time (*τ_lag_*) for the aggregation reactions [80,81,82] were calculated by fitting kinetic traces for ThT fluorescence and Rayleigh light scattering (RLS).

### 4.7. Time-Resolved Fluorescence Measurements

Fluorescence decay curves were recorded with a spectrometer, FluoTime 300 (PicoQuant, Berlin/Heidelberg, Germany) with a Laser Diode Head LDH-C-440 (λ_ex_ = 440 nm). The fluorescence of ThT was registered at λ_em_ = 490 nm. The fluorescence lifetime of ThT bound to studied aggregates was calculated using recorded fluorescence decay curves. For this, the measured emission decays were fit to a multiexponential function using the standard convolute-and-compare nonlinear least-squares procedure [83]. In this method, the convolution of the model exponential function with the instrument response function (IRF) was compared to the experimental data until a satisfactory fit was obtained. The fitting routine was based on the nonlinear least-squares method. Minimization was performed according to Marquardt [84].

### 4.8. Confocal Microscopy

For obtaining the fluorescence images of the ThT-stained amyloid structures, the confocal laser scanning microscope Olympus FV 3000 (Olympus, Tokyo, Japan) was used. We used the oil immersion objective with a 60× magnification, numerical aperture NA 1.42 and laser with an excitation line of 405 nm.

### 4.9. SDS-PAGE

The pseudo-native SDS-PAGE analysis of the same supernatants and pellets was performed with 17% polyacrylamide gel (0.375 M Tris HCl, pH 8.8, 0.1% SDS). Samples were loaded on the gel in a buffer containing 0.0625 M Tris HCl, pH 6.8, 1% SDS, 10% glycerol without boiling [85]. To estimate the proportion of the monomer fraction in the samples, the SDS-PAGE results were analyzed using ImageJ software. Data for the samples with vicilin amyloids were normalized to the area of the band of control samples.

### 4.10. Study of Interaction between Vicilin Amyloids and Mammalian Proteins in Different Forms

To demonstrate the direct interaction of mammalian proteins in monomeric and aggregated forms with vicilin amyloids, a 0.5 mg/mL concentration of mammalian proteins and 0.1 mg/mL concentration of vicilin fibrils were mixed, incubated for 2 h and analyzed with far-UV CD spectroscopy. The concentration of vicilin fibrils was chosen so that they did not contribute to the recorded CD spectra. Samples containing mammalian proteins and vicilin amyloids separately at the same concentrations were used as controls. The interaction of different forms of the lysozyme with vicilin amyloids was detected with the change in the recorded CD spectrum of the samples after the addition of the latter (as it was made in [32]). The formation of a complex between a monomeric lysozyme and vicilin amyloids was also analyzed for the same samples by measuring intrinsic lysozyme fluorescence (because of the presence of Trp residues in this protein structure). The integrated fluorescence intensity (*F_total_*), wavelength of the fluorescence spectrum maximum (λ*_max_*), parameter *A* (which is the ratio of the fluorescence intensities of the sample at two wavelengths—320 and 365 nm—and is sensitive to changes in the position and shape of fluorescence spectra [33,34]) and fluorescence anisotropy (*r*) were determined.

### 4.11. Cell Viability Assessment

Cell viability was analyzed using cervical cancer (HeLa) and gastric adenocarcinoma (AGS) human cell lines; cells were within the first 13 passages when the experiment was conducted. Cell lines were obtained from the shared research facility “Vertebrate cell culture collection”. The toxicity was assessed with the MTT (Sigma-Aldrich, USA) reduction inhibition assay based on the protocol described for the first time by Mosmann [86]. Briefly, cells were routinely cultured in DMEM–10%-FBS supplemented with 2 mM of l-glutamine and kept in a 5% CO_2_ humidified incubator at 37 °C. For the assay of the fibrils’ influence on the cell’s vitality, cells (60% confluence) were stripped from culture flasks with 0.25% Trypsin-EDTA, washed with DPBS and plated in 96-well coated culture plates at a density of 5000 viable cells/well in 100 µL of DMEM. The cells were incubated at 37 °C and 5% CO_2_ for 24 h and amyloid aggregates were administered to cells in a concentration of 0.014 mg/mL. After 24 h, DMEM was removed and cells were incubated for 3 h with 100 μL of DMEM without phenol red and FBS, containing 0.5 µg/µL of MTT. Following the treatment, 100 μL of DMSO was added to each well and the samples were incubated at 37 °C to allow complete lysis. The absorbance values were determined at 595 nm with an automatic plate reader (Bio-Rad, Milan, Italy). The final absorption values were calculated by averaging 5 independent measurements of each sample and subtracting the average of the blank from this. Controls contained equal amounts of an incubation buffer.

All experiments were performed at least in triplicate. To test the sample data for normal distribution, the Kolmogorov–Smirnov test was used. Multiple group comparisons were processed using the one-way analysis of variance (ANOVA) method with Tukey’s post hoc test. The differences were considered significant at *p* < 0.05. Data were analyzed using online calculator software (https://astatsa.com/OneWay_Anova_with_TukeyHSD/, accessed on 1 January 2023).

The toxicity of the fibrils against the monocytic leukemia cell line THP-1 (ATCC TIB-202) was assessed as follows. The cells were plated in 96-well culture plates in 100 μL of an RPMI medium supplemented with 10% FBS, 50 μg/mL of gentamycin, 0.05 mM of β-mercaptoethanol and a 10% fibril buffer at a density of 5 × 10^5^ cells/mL. The final concentration of fibrils was 0.01 mg/mL. Cells were incubated at 37 °C and 5% CO_2_ for 24 h or 48 h. Then, the cells’ viability was tested according to the following protocol [87]. Briefly, 10 μL of the MTT solution in PBS (5 mg/mL) was added to each well and the plate was incubated at 37 °C and 5% CO_2_. After 4 h, 100μL of the SDS-HCl solution (10% SDS and 0.01 N HCl) was added and incubated for another 18 h. The optical density at 570 nm was measured and subtracted by the optical density at 620 nm.

The experiments were performed in four replicates. Multiple group comparisons were processed using the one-way analysis of variance (ANOVA) method with the emmeans post hoc test (emmeans R package, https://github.com/rvlenth/emmeans, accessed on 1 January 2023). The differences were considered significant at *p* < 0.05.

### 4.12. Statistical Analysis

The photophysical characteristics of amyloids and bound-to-fibrils ThT were determined based on the results of at least three independent experiments. The standard error of the mean was determined for a confidence interval of 0.95. The reliability of the results was verified according to the Mann–Whitney U test or one-way analysis of variance (ANOVA) using Graphpad Prism (Version 9.1.0) software.

## Figures and Tables

**Table 1:** Properties of amyloid fibrils formed from mammalian proteins in the absence and in the presence of vicilin amyloids and photophysical properties of ThT bound to fibrils.

Amyloidogenic Protein/Peptide	Vicilin Amyloids	RLS	Turbidity	β-Sheets, %	*F_ThT_*·10^−4^	*q_ThT_*·10^2^	*<τ_ThT_>*, ns
Lysozyme	–	423 ± 16	0.53 ± 0.02	45.6 ± 1.4	12.2 ± 0.3	2.2 ± 0.1	1.1 ± 0.1
	+	81 ± 6	0.26 ± 0.01	37.1 ± 1.2	5.0 ± 0.4	2.2 ± 0.2	1.1 ± 0.1
β2m	–	233 ± 9	0.24 ± 0.01	35.3 ± 1.2	4.3 ± 0.2	4.6 ± 0.2	1.6 ± 0.1
	+	209 ± 7	0.21 ± 0.02	35.2 ± 1.3	3.9 ± 0.1	4.8 ± 0.1	1.6 ± 0.1
Aβ42	–	399 ± 12	0.78 ± 0.02	42.2 ± 1.5	8.1 ± 0.2	1.3 ± 0.2	0.8 ± 0.1
	+	267 ± 12	0.61 ± 0.03	40.8 ± 1.0	7.1 ± 0.2	1.3 ± 0.2	0.8 ± 0.1
Insulin	–	460 ± 9	3.13 ± 0.04	39.3 ± 1.2	6.2 ± 0.1	2.2 ± 0.1	0.8 ± 0.1
	+	422 ± 8	2.52 ± 0.07	33.7 ± 1.2	15.0 ± 0.3	1.4 ± 0.1	1.0 ± 0.1

RLS*—*Rayleigh light scattering of the samples, *F_ThT_*, *q_ThT_*, *τ_ThT_—*fluorescence intensity, quantum yield and lifetime of ThT bound to amyloids.

## Data Availability

The data presented in this study are within the paper and supplementary materials.

## Supplementary Materials

The following supporting information can be downloaded at: https://www.mdpi.com/article/10.3390/ijms241612932/s1.
