# Supplementary material for: Amyloid Fibrils of Pisum sativum L. Vicilin Inhibit Pathological Aggregation of Mammalian Proteins
Source: Int J Mol Sci. 2023 Aug 18;24(16):12932. doi: 10.3390/ijms241612932 (PMC10454621; doi:10.3390/ijms241612932)
Supplement: Supplementary file 1 [file ijms-24-12932-s001.zip › ijms-2539575-supplementary.pdf]

Supplementary Materials

Amyloid fibrils of *Pisum sativum* L. vicilin inhibit pathological aggregation of mammalian proteins

Maksim I. Sulatsky[1], Mikhail V. Belousov[2,3], Anastasiia O. Kosolapova[2,3], Ekaterina V. Mikhailova[1], Maria N. Romanenko[2,3], Kirill S. Antonets[2,3], Irina M. Kuznetsova[1], Konstantin K. Turoverov[1], Anton A. Nizhnikov[2,3,*], Anna I. Sulatskaya[1,*]

[1]Institute of Cytology, Russian Academy of Sciences, St. Petersburg, Russia
[2]All-Russia Research Institute for Agricultural Microbiology, St. Petersburg, Russia
[3]Faculty of Biology, St. Petersburg State University, St. Petersburg, Russia
* Correspondence: Anton A. Nizhnikov, a.nizhnikov@arriam.ru or a.nizhnikov@spbu.ru; Anna I. Sulatskaya, ansul@mail.ru

Supplementary Table S1. Fibrillation kinetics parameters of amyloid fibrils formed from lysozyme in the absence and in the presence of vicilin, cupin-1.1 and cupin-1.2 amyloids.

**Sample**	$K_{app} \times 10^{-2}$, h^{-1}		τ_{lag}, h	
	**ThT**	**RLS**	**ThT**	**RLS**
lysozyme	1.5±0.1	1.5±0.1	242±21	225±20
lysozyme_vicilin	0.9±0.2	1.1±0.2	239±45	278±41
lysozyme _cupin-1.1	1.5±0.2	1.5±0.1	284±24	288±27
lysozyme _cupin-1.2	1.6±0.2	1.4±0.2	277±31	296±31

K_{app} – apparent rate constant and τ_{lag} – lag time of fibrillogenesis.

Supplementary Table S2. Characteristics of monomeric lysozyme in the absence and in the presence of vicilin amyloids.

**Sample**	F_{total}	λ_{max}, nm	A	r
lysozyme	(3.8±0.1)*10^4	333 ± 1	1.48±0.01	0.15±0.01
lysozyme + vicilin	(3.7±0.1)*10^4	334 ± 1	1.47±0.01	0.15±0.01

F_{total} - integrated fluorescence intensity, λ_{max} - wavelength of fluorescence spectrum maximum, A - parameter A (the ratio of fluorescence intensities at two wavelengths - 320 and 365 nm) and r - fluorescence anisotropy.

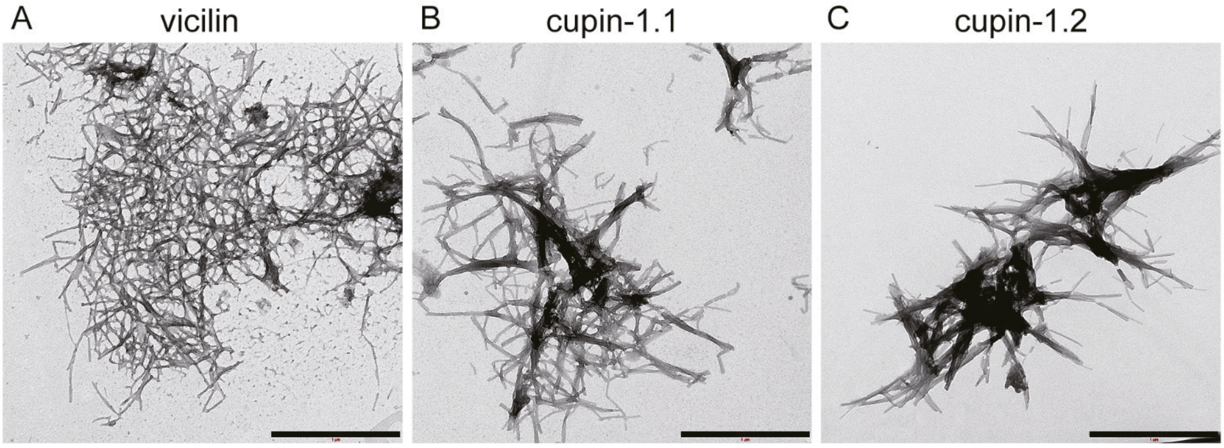

Supplementary Figure S1. Amyloid fibrils formed from vicilin and its fragments. TEM images of fibrils formed by (A) vicilin, (B) cupin-1.1 and (B) cupin-1.2. Scale bars correspond to 1 μm.

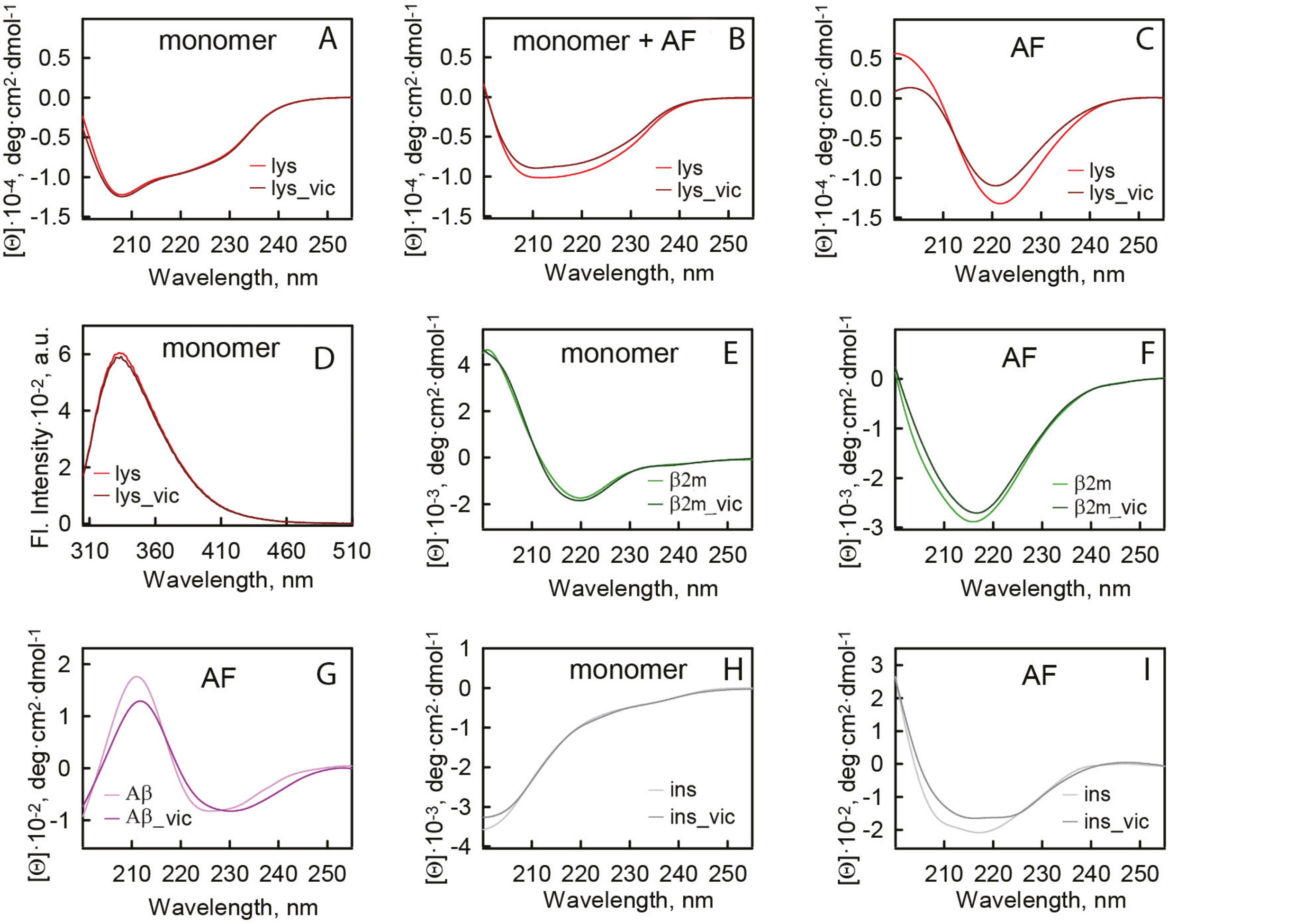

Supplementary Figure S2. Analysis of a direct interaction between vicilin amyloids and mammalian proteins in different forms. (A-C, E-I) Far-UV CD spectra of (A, E, H) native monomeric, (B) different amyloidogenic forms during fibrillogenesis and (C, F, G, I) mature amyloids of (A-C) lysozyme, (E-F) β2m, (G) Aβ42, and (H-I) insulin. (D) Fluorescence spectra of native monomeric lysozyme. Spectra of probes without (lys, β2m, Aβ42, and ins) and with (lys_vic, β2m_vic, Aβ42_vic, and ins_vic) vicilin fibrils are presented. Vicilin fibrils did not contribute to the recorded CD and fluorescence spectra. The interaction of various forms of mammalian proteins with vicilin amyloids could be detected by the change in their CD and fluorescence spectra of the sample after the addition of the latter.

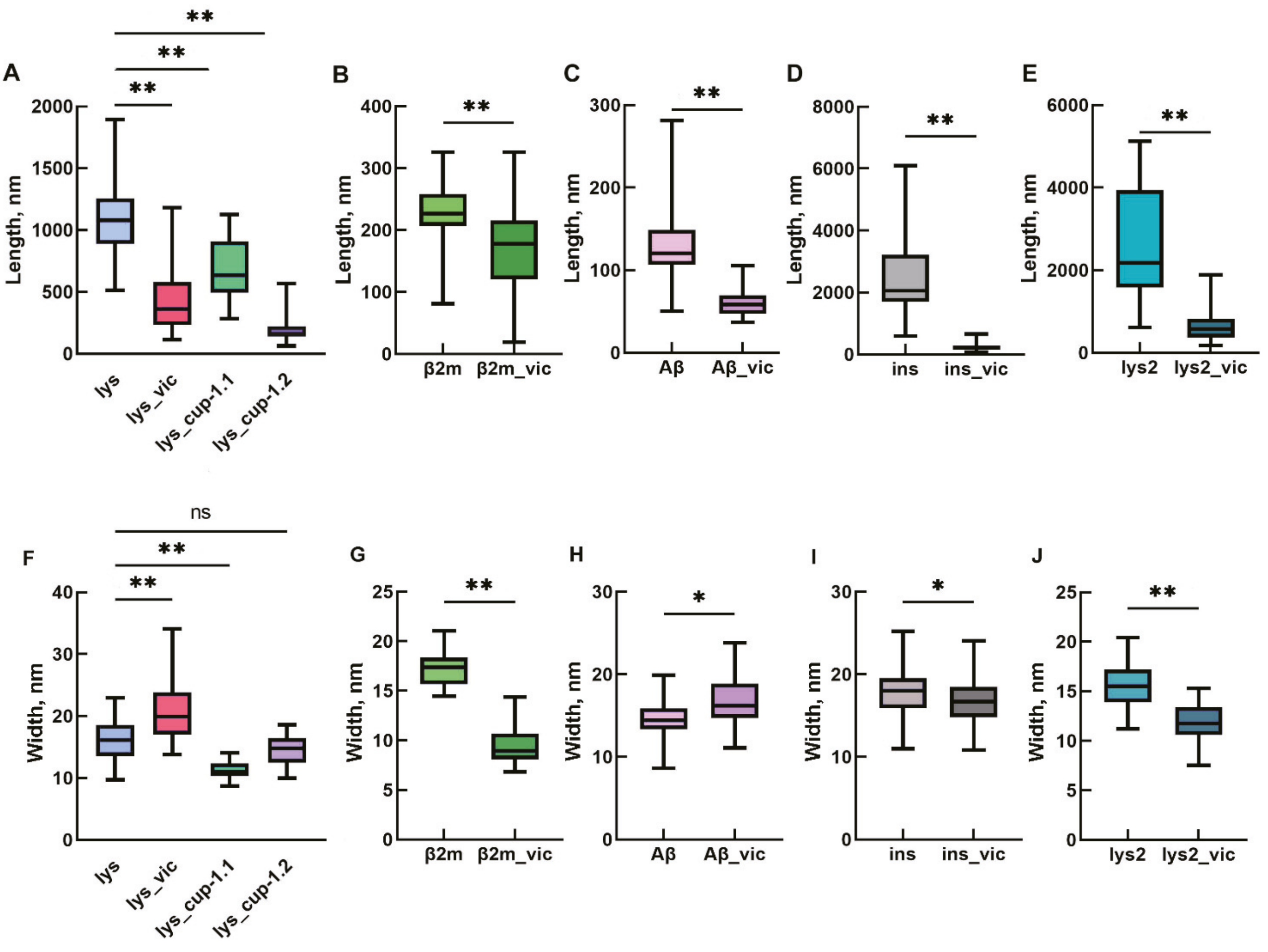

Supplementary Figure S3. Assessment of changes in the length and width of fibrils in the samples with lysozyme ((A, F) lys and (E,J) lys2 – for slow and fast kinetics, respectively), (B, G) β2m, (C,H) Aβ42 (Aβ), and (D, I) insulin (ins) after vicilin amyloids exposure (lys_vic, etc.) using the analysis of TEM data by ImageJ software. Data are presented as the minimum and maximum, the sample median, and the interquartile range for a few dozen fibrils (from 50 to 150) for each sample. * $p \leq 0.05$, ** $p \leq 0.01$.

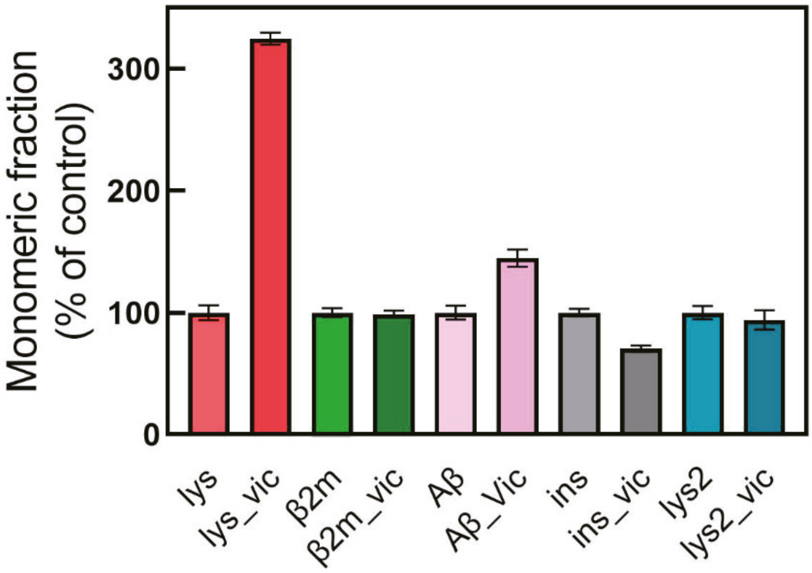

Supplementary Figure S4. Assessment of changes in the monomeric fraction content in the samples with lysozyme (lys and lys2 – for slow and fast kinetics, respectively), β2m, Aβ42 (Aβ), and insulin (ins) after vicilin amyloids exposure (lys_vic, etc.) using the analysis of the bands on the SDS-PAGE by ImageJ software. Data were normalized to the area of the bands in control samples.

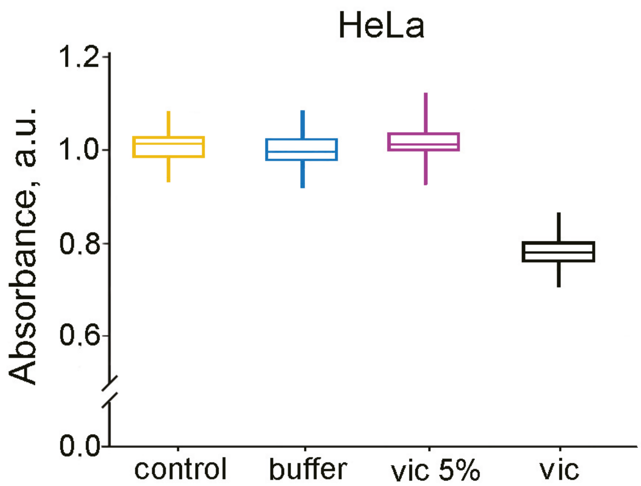

Supplementary Figure S5. The data of MTT assay for evaluation of the metabolic activity of HeLa cell line. Cells were exposed to vicilin amyloids in concentrations in which they were added to the samples with mammalian proteins (vic 5%) and equal to the concentration of mammalian proteins in the samples (vic) for 24 hours. Data are given as the minimum and maximum, the sample median, and the interquartile range for triplicate samples.
